# The Divergent Effects of Fear and Disgust on Inhibitory Control: An ERP Study

**DOI:** 10.1371/journal.pone.0128932

**Published:** 2015-06-01

**Authors:** Mengsi Xu, Zhiai Li, Cody Ding, Junhua Zhang, Lingxia Fan, Liuting Diao, Dong Yang

**Affiliations:** 1 School of Psychology, Southwest University, Chongqing, China; 2 Key Laboratory of Cognition and Personality (Southwest University), Ministry of Education, Chongqing, China; 3 The School of Psychology and Cognitive Science, East China Normal University, Shanghai, China; 4 University of Missouri-St. Louis, St. Louis, MO, United States of America; 5 Center for Psychological Application, Department of Psychology, South China Normal University, Guangzhou, China; Anhui Medical University, CHINA

## Abstract

Negative emotional stimuli have been shown to attract attention and impair executive control. However, two different types of unpleasant stimuli, fearful and disgusting, are often inappropriately treated as a single category in the literature on inhibitory control. Therefore, the present study aimed to investigate the divergent effects of fearful and disgusting distracters on inhibitory control (both conscious and unconscious inhibition). Specifically, participants were engaged in a masked Go/No-Go task superimposed on fearful, disgusting, or neutral emotional contexts, while event-related potentials were measured concurrently. The results showed that for both conscious and unconscious conditions, disgusting stimuli elicited a larger P2 than fearful ones, and the difference waves of P3 amplitude under disgusting contexts were smaller than that under fearful contexts. These results suggest that disgusting distracters consume more attentional resources and therefore impair subsequent inhibitory control to a greater extent. This study is the first to provide electrophysiological evidence that fear and disgust differently affect inhibitory control. These results expand our understanding of the relationship between emotions and inhibitory control.

## Introduction

Many studies have demonstrated that negative emotional stimuli impair executive control [1,2]. For example, Hartikainen et al. [1] showed that presenting threat-related images (e.g. an image of a spider) increases the no-go error rate in a Go/No-Go task. Similarly, using the stop-signal task, Verbruggen and De Houwer [2] found that negative emotional stimuli (e.g. mutilation pictures) interrupt on-going task performance. Most often, this impairment is explained by the assumption that negative stimuli draw attention and receive preferential processing because of their potentially threatening nature, thus conferring an evolutionary advantage [3]. An important issue is that in these studies, all the negative stimuli were treated as a single category. This may be problematic because different types of negative stimuli (e.g. fearful and disgusting) may convey divergent information [4].

Although fear and disgust are two emotions that share a similar emotional valence and level of arousal, they are distinct in many aspects [4]. First, their biological meaning is different. Fear generally signals an immediate threat to survival [5], while disgust is primarily related to contamination and represents a possible danger [6]. Second, their cognitive function is divergent. Fear enhances sensory acquisition in order to facilitate a quick response, but disgust works in the opposite fashion by diminishing environmental input to avoid contamination [7,8]. Third, their demand for exploration is heterogeneous [9]: Although disgusting stimuli might pose a potential danger, there is no biological cost associated with their exploration; in contrast, more costs than benefits are found when directing resources to explore fearful stimuli. Consequently, it is more beneficial to allocate resources toward exploring disgusting stimuli than it is toward exploring fearful stimuli.

Indeed, several studies have demonstrated that fear and disgust exert different effects on perception, attention, and memory [7,9,10]. For instance, Carretié et al. [9] asked participants to perform a digit categorization task under different emotional contexts (fearful, disgusting, and neutral) and found that responses during the disgusting contexts were slower than were those during fearful contexts. However, whether and how fear and disgust distinctly affect executive functions such as inhibitory control remains unclear.

Inhibitory control, the ability to suppress inappropriate behaviours, is an essential component of executive control. Traditionally, inhibitory control has been viewed as a process that depends on the conscious detection of response-relevant signals [11]. However, recent research using a masked Go/No-Go task has shown that inhibitory control can be triggered unconsciously [12]. Specifically, van Gaal et al. [12] developed a masked Go/No-Go task that included weakly (conscious) and strongly masked (unconscious) go/no-go trials. In the weakly masked condition, a go or no-go prime was presented for 233 ms, followed by a metacontrast masking annulus presented for 17 ms. In the strongly masked condition, the durations of the go or no-go prime and annulus were 17 ms and 233 ms, respectively. Participants are instructed to respond as fast as possible to the annulus (response signal) but withhold their response when a no-go signal briefly precedes the annulus. However, when a go signal precedes the annulus, they are instructed to respond as quickly as possible [12]. Consequently, in the weakly masked condition, participants would respond similar to the traditional Go/No-Go task (i.e. they consciously perceived the go/no-go signal). While in the strongly masked condition, participants would make a go response to the strongly masked no-go trials because the no-go signal could not be perceived consciously (therefore the RT for unconscious no-go trials can be directly measured). Furthermore, the RT slowing (i.e. mean RT for strongly masked no-go trials minus strongly masked go trials) could be used as an index of unconscious inhibitory control because it was positively correlated with the neural activation magnitude of the unconscious inhibition network [12–14]. Using this masked Go/No-Go task, van Gaal et al. [12] found that the responses in the strongly masked no-go trials were significantly longer than were those in the strongly masked go trials. These results confirmed the existence of unconscious inhibitory control.

Several studies have investigated the relationship between unconsciously and consciously triggered inhibitory control and have indicated that they are strongly related [12,15]. On one hand, unconscious and conscious inhibitory control can similarly activate prefrontal control networks [12,16]. On the other hand, unconscious and conscious inhibitory controls differ in the strength, duration, and scope of neural activity [15]. Recent studies on conflict adaptation [17] and linguistic operation [18] have demonstrated that unconscious and conscious executive control are related. For example, Desender et al. [17] used a masked priming paradigm to examine conflict adaptation. They observed conflict adaptation in both unconscious and conscious conditions, but found that the conflict adaptation effect was smaller in the unconscious condition than it was in the conscious condition. Therefore, it appears that while unconscious and conscious inhibitory control differs in the degree of information processing, they might be similar in many aspects.

Therefore, although previous work has investigated the divergent effects of fear and disgust on perception, attention, and memory, little is known about the differential effects of fear and disgust on inhibitory control, not to mention whether these effects are similar for conscious and unconscious inhibition. As inhibitory control processes are important in daily life (e.g. inhibitory control likely helps many of us avoid embarrassing or socially inappropriate situations and might be crucial for adaptive goal-directed behaviour [12,19]), it is necessary to explore whether different emotions (e.g. fear and disgust) exert divergent impacts on inhibitory control.

Due to their high temporal resolution, event-related potentials (ERPs) are particularly well suited to studying emotion-modulated response inhibition [20]. In affective science, researchers have pointed out that P2, a positive peaking potential between 180 and 350 ms which is typically located over the centro-parietal and parieto-occipital regions [21], shows significant amplitude increments when a negative stimulus automatically attracts attention in a wide variety of tasks [9,22]. For instance, Carretié et al. [9] observed larger P2 for disgusting stimuli than for neutral ones, more importantly, they also observed larger P2 for disgusting stimuli than for fearful ones. These results indicated that disgusting distracters were more efficient at attracting attention. In addition, previous studies on response inhibition have confirmed that the Go/No-Go paradigm (both traditional and masked Go/No-Go task) typically elicits a centro-parietal P3 (300–600 ms), which has larger amplitudes in no-go trials than in go trials [23]. The difference waves of P3 amplitude (no-go condition minus go condition) can be used as an index of inhibiting ability, with larger P3 difference waves representing a stronger ability to suppress the prepotent response [24].

In sum, the current study aimed to investigate whether fear and disgust exert different effects on inhibitory control, and whether these effects are similar for conscious and unconscious inhibitory control. Specifically, participants were asked to perform a masked Go/No-Go task in fearful, disgusting, or neutral emotional contexts. ERPs were measured concurrently. Building on previous studies, we hypothesized that fear and disgust has distinct effects on attention and inhibitory control. On one hand, previous studies have shown that disgusting stimuli attracted more attention than fearful ones [9]. On the other hand, cognitive resources are limited [25]. Therefore, when limited attention is diverted to aid in the processing of disgusting stimuli, the subsequent inhibitory control might be impaired [25,26]. Specifically, at the behavioural level, given that the RT slowing was used as an index of inhibiting ability, we hypothesized that the RT slowing under disgusting contexts would be smaller than that under fearful contexts. At the neural level, given that P2 acts as an index of attention allocation [9], we hypothesized that disgusting distracters would elicit larger P2 than fearful ones would; and given that the difference wave of P3 is an index of inhibiting ability [12,16], we hypothesized that this difference would be smaller under disgusting contexts compared to that under fearful contexts. Furthermore, as some researchers have indicated that conscious and unconscious inhibitory control are similar in many aspects [12,15], we hypothesized that the effects of fear and disgust on conscious and unconscious inhibition might be similar.

## Methods

### Ethics statement

The ethics committee of Southwest University of China approved this experiment. Written informed consent was obtained from all participants in compliance with the principles of the Declaration of Helsinki.

### Participants

A total of 18 female university students aged 18–24 years (*M* = 20.11 years, *SD* = 1.23) participated in the study. All participants were right-handed with normal or corrected-to-normal vision. Upon completion of the task, they received 30 RMB for their participation. We selected only women for this experiment because previous studies have shown that women display greater vigilance to emotional stimuli [27]; therefore, using an all-female sample removed gender as a confounding variable.

### Stimulus selection

The stimuli consisted of three geometric figures and 30 emotional images. The geometric figures consisted of an annulus (visual angle of 0.8°), a square (visual angle of 0.47° × 0.47°), and a diamond (the same square tilted by 45°), filled with green colour and outlined in black. Therefore, they were clearly differentiated from the background images on which they were superimposed [9].

Thirty emotional pictures were selected to generate different background contexts (10 fearful, 10 disgusting, and 10 neutral). The size of all the pictures was 7.26° (width) × 4.53° (height). They were taken from the International Affective Picture System (IAPS) [28] and EmoMadrid (Emotional Picture Database: http://www.uam.es/carretie/EmoMadrid.htm) and conveyed fearful (e.g. snakes, guns), disgusting (e.g. faeces, vomit), or neutral content (e.g. tables, cups). These images were selected according to objective criteria (valence and arousal assessments, which were similar for fear and disgust categories) and subjective criteria (trying to select “pure” fearful and disgusting pictures, which meant fearful pictures conveyed more fearfulness than did disgusting and neutral pictures, and disgusting pictures conveyed more disgustingness than did fearful and neutral pictures). Twenty-six independent judges (all women, 18–24 years) were asked to assess each picture and rate them from 1–9 on valence (1 = *negative*, 9 = *positive*), arousal (1 = *calming*, 9 = *arousing*), fearfulness (1 = *minimum*, 9 = *maximum*), and disgustingness (1 = *minimum*, 9 = *maximum*) (see Table 1).

**Table 1:** Subjective Ratings of Stimuli and Behavioural Results for Go/No-Go Stimuli.

	Neutral	Fear	Disgust
	*M* (*SD)*	*M* (*SD)*	*M* (*SD)*
Subjective ratings			
Valence	5.41 (0.65)	2.11 (0.88)	1.85 (0.79)
Arousal	2.44 (1.45)	7.13 (1.28)	7.15 (1.73)
Disgustingness	2.23 (1.41)	5.82 (1.40)	7.72 (1.63)
Fearfulness	1.62 (0.83)	7.38 (0.94)	4.80 (2.11)
Behaviour results			
Weakly masked trials			
Go RT	523.39 (53.39)	523.53 (49.31)	534.71 (48.52)
Go accuracy	0.99 (0.01)	0.99 (0.01)	0.99 (0.01)
No-go accuracy	0.92 (0.08)	0.93 (0.09)	0.93 (0.07)
Strongly masked trials			
Go RT	468.29 (52.59)	466.15 (46.31)	464.83 (53.00)
No-go RT	475.91 (46.51)	478.03 (55.85)	472.03 (50.60)
Go accuracy	0.99 (0.02)	1.00 (0.00)	1.00 (0.00)
No-go accuracy	0.99 (0.02)	0.99 (0.01)	0.99 (0.01)

RT, reaction time.

**Fig 1:**
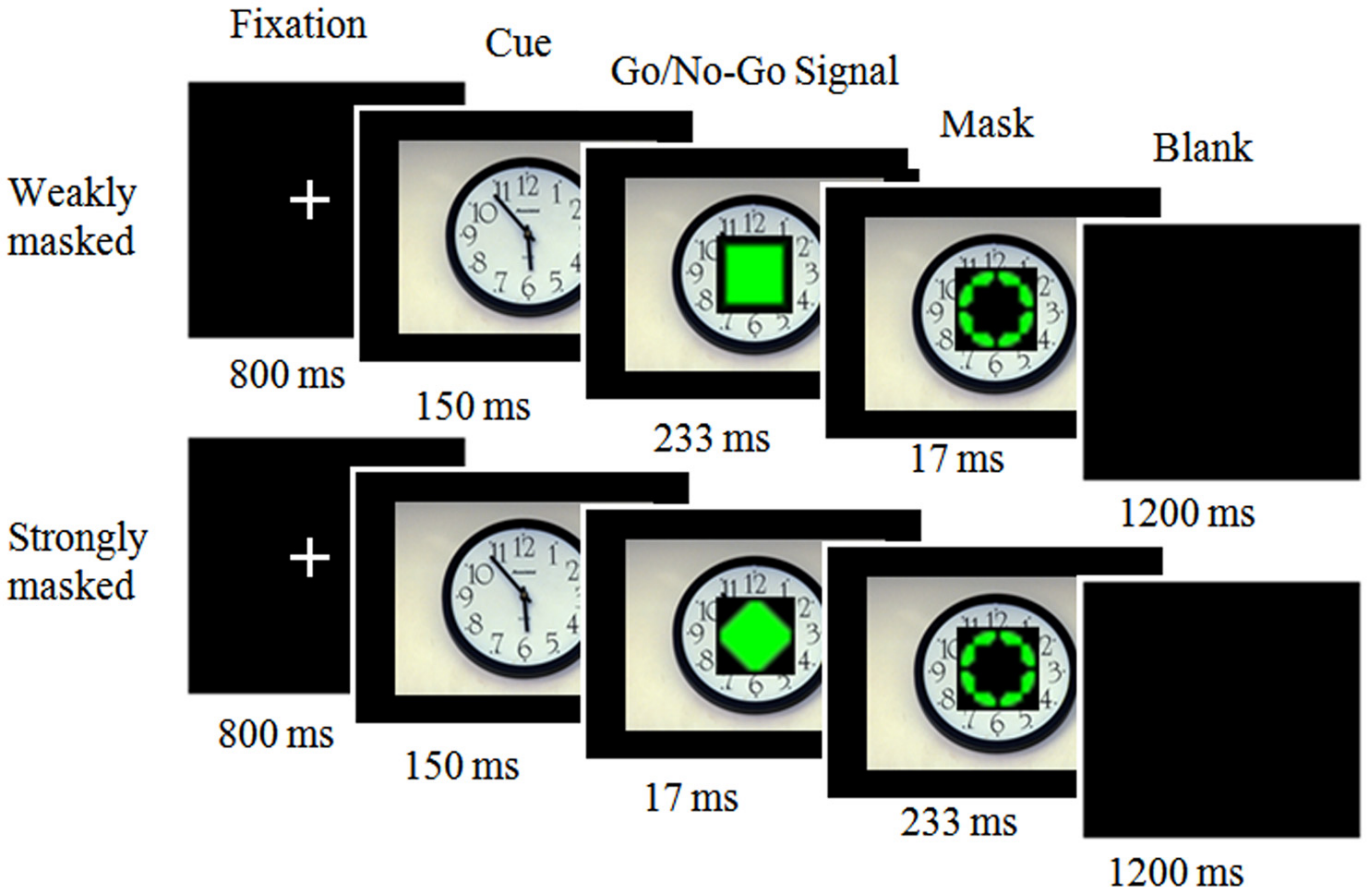
Procedure and design of Experiments. Participants were engaged in a masked Go/No-Go task superimposed on fearful, disgusting, or neutral emotional contexts.

A one-way ANOVA and *t*-test on the assessments showed that fearfulness was significantly higher for the fearful pictures than it was for the disgusting and neutral pictures, *t* (25) = 6.61, *SD* = 1.99, *p* < 0.01; *t* (25) = 21.88, *SD* = 1.34, *p* < 0.01, respectively. Disgustingness was significantly higher for the disgusting pictures than it was for fearful and neutral pictures, *t* (25) = 5.01, *SD* = 1.92, *p* < 0.01; *t* (25) = 11.90, *SD* = 2.35, *p* < 0.01, respectively. Valence was lower for the fearful and disgusting pictures than it was for the neutral pictures, *t* (25) = -15.41, *SD* = 1.09, *p* < 0.01; *t* (25) = -18.59, *SD* = 0.98, *p* < 0.01, while arousal was higher for fearful and disgusting pictures than it was for neutral pictures, *t* (25) = 12.59, *SD* = 1.90, *p* < 0.01; *t* (25) = 10.48, *SD* = 2.28, *p* < 0.01, respectively. Moreover, there was no significant difference between the fearful and disgusting pictures in terms of valence and arousal, valence: *t* (25) = 1.66, *SD* = 0.78, *p =* 0.11; arousal: *t* (25) = -0.12, *SD* = 0.94, *p* = 0.91. Therefore, we chose fearful pictures that represented more fearfulness than did disgusting and neutral pictures and disgusting pictures that represented more disgustingness than did fearful and neutral pictures, while keeping their valence and arousal ratings constant. Furthermore, since the complexity of images would affect early visual ERPs [29], only simple figure-ground images (these images have a relatively clear figure-ground composition, just like the clock in Fig 1) were chosen to remove the image complexity as a confounding variable.

All stimuli were displayed on a Dell computer with a 20-inch monitor (60-Hz refresh rate). E-prime software (Psychology Software Tools, Inc., Sharpsburg, PA) was used to present the stimuli and collect data.

### Design and procedure

The experiment consisted of two phases: the masked Go/No-Go task and the awareness test.

#### The masked Go/No-Go task

Subjects were placed in an electrically shielded, soundproofed room. They were asked to perform the masked Go/No-Go task superimposed on an emotional image (Fig 1).

Each trial began with a crosshair presented in the centre of the screen (800 ms), followed by the emotional image centrally displayed for 150 ms. Next, the masked Go/No-Go task was superimposed on the emotional image (250 ms). Finally, a blank screen was presented for 1200 ms. The masked Go/No-Go task was adapted from van Gaal et al. [12] and consisted of weakly and strongly masked go/no-go trials. In the weakly masked condition, a go or no-go prime was presented for 233 ms, followed by a metacontrast masking annulus presented for 17 ms. In the strongly masked condition, the durations of the go or no-go prime and the metacontrast masking annulus were 17 ms and 233 ms, respectively. We used an annulus as a metacontrast mask because it strongly reduces stimulus visibility [30]. The stimulus parameters were akin to those reported by van Gaal et al. [12].

Subjects viewed the display from a fixed distance of 60 cm and were instructed to respond to a green annulus (response signal under strongly masked condition) as quickly as possible by pressing the “M” key on a standard keyboard with their right index finger but withhold their response when a green diamond (no-go signal) preceded the annulus. However, when a green square (go signal) preceded the annulus, they were instructed to respond as quickly as possible by pressing the “M” key.

There were five blocks and each contained 120 trials, for a total of 600 trials (150 trials for each condition). The presentation of the go/no-go signals and emotional cues were displayed in a random order. The stimulus used as the no-go signal (square or diamond) was counterbalanced across subjects.

Before the experiment began, participants completed a practice block of 40 trials with 10 neutral images as the background; this was done to ensure that they understood the task instructions. These neutral images were different from those used in the formal experiment.

#### The awareness test

The prerequisite of our study was the effectiveness of our masking procedure. Therefore, to assess whether participants were truly unaware of the strongly masked prime, an alternative forced-choice discrimination task was added, which included 80 strongly masked trials after the formal task. Participants were asked to press “F” or “J” depending on the shape (square or diamond) that flashed before the annulus in the trial. They were informed that response time was not important and were asked to respond as accurately as possible.

### EEG recordings

Brain electrical activity was recorded at 64 scalp sites using tin electrodes mounted in an elastic cap (Brain Product, Munich, Germany), with references on the left and right mastoids, and a ground electrode on the medial frontal aspect. The vertical electrooculograms (EOGs) were recorded supra- and infra-orbitally at the right eye. The horizontal EOG was recorded from the left versus the right orbital rim. The EEG and EOGs were amplified using a 0.05–100 Hz bandpass and continuously digitized at 500 Hz/channel. All inter-electrode impedance was maintained below 5 kΩ.

### Data analysis

#### Behavioural analysis

For the awareness test, a one-sample t-test was performed on the *d’* scores (tested against 0). For the masked Go/No-Go task, the key dependent variables were accuracy and RT under different emotional contexts. Only correct responses between 100 and 1200 ms were analysed. For the strongly masked condition, accuracy and RTs were separately analysed using an emotion (fear, disgust, neutral) × prime type (strongly masked go trial vs. strongly masked no-go trial) within-subjects ANOVA, and then the RT slowing under fear, disgust, and neutral contexts was analysed using one-way ANOVA. For the weakly masked condition, accuracy for go/no-go trials was first analysed using an emotion (fear, disgust, neutral) × prime type (weakly masked go trial vs. weakly masked no-go trial) within-subjects ANOVA, and RTs for go trials under fearful, disgusting, and neutral emotional contexts were analysed using a one-way ANOVA.

#### ERP analysis

The data were referenced to the average of the left and right mastoids (average mastoid reference), and a bandpass filter of 0.3–40 Hz was applied. Eye movement artefacts (such as eye movements and blinking) were excluded offline. Trials contaminated with artefacts due to amplifier clipping and peak-to-peak deflection exceeding ±70 μV were excluded from the average. Only trials with correct responses were analysed. The continuous recording was divided into 800-ms epochs for each trial, beginning 100 ms before the go/no-go signal onset.

**Fig 2:**
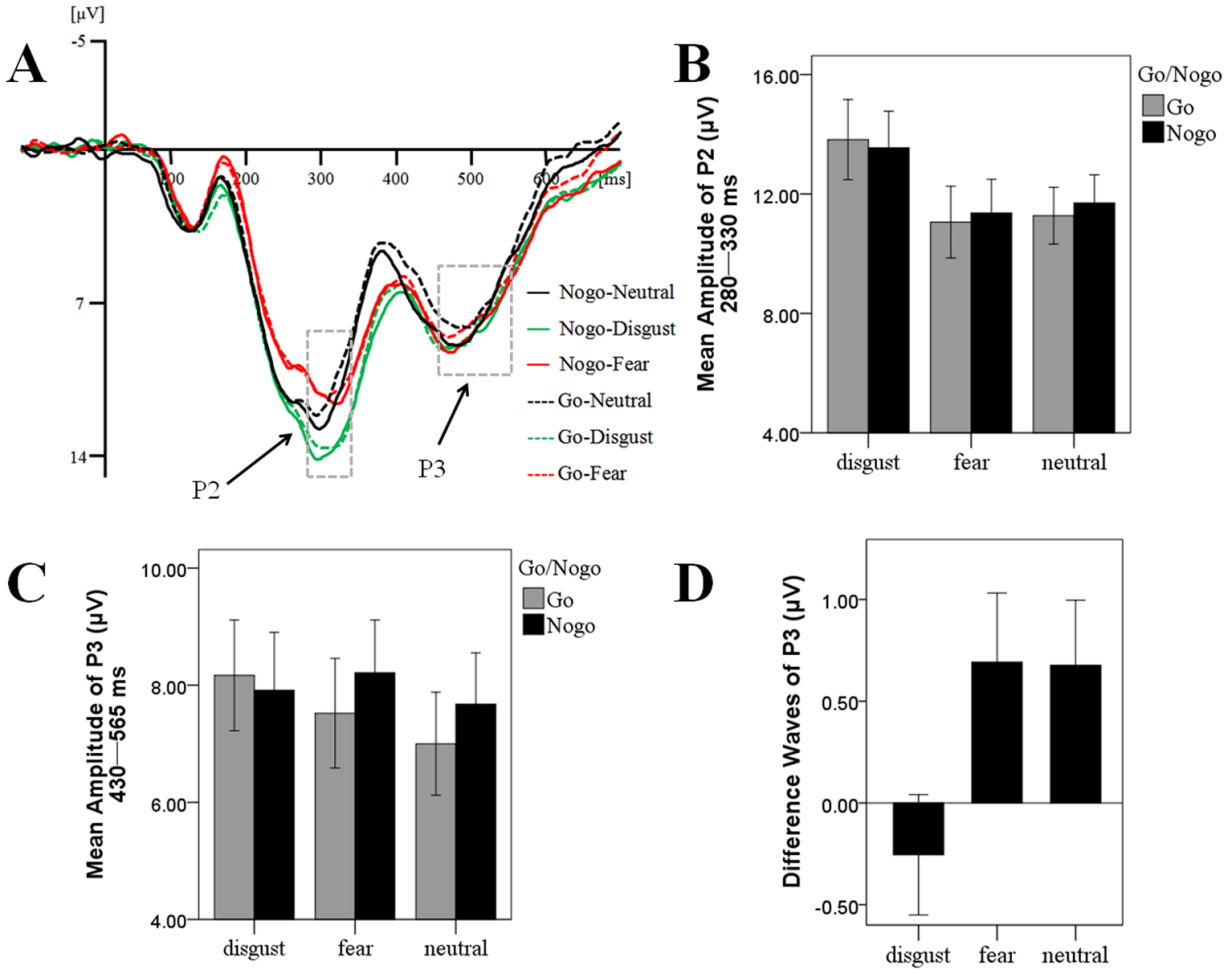
The ERP Results for Strongly Masked Condition. (A) The averaged ERP under different emotional contexts; (B) P2 amplitude under different emotional contexts; (C) P3 amplitude under different emotional contexts; (D) The difference waves (no-go condition minus go condition) of P3 amplitude under different emotional contexts.

**Fig 3:**
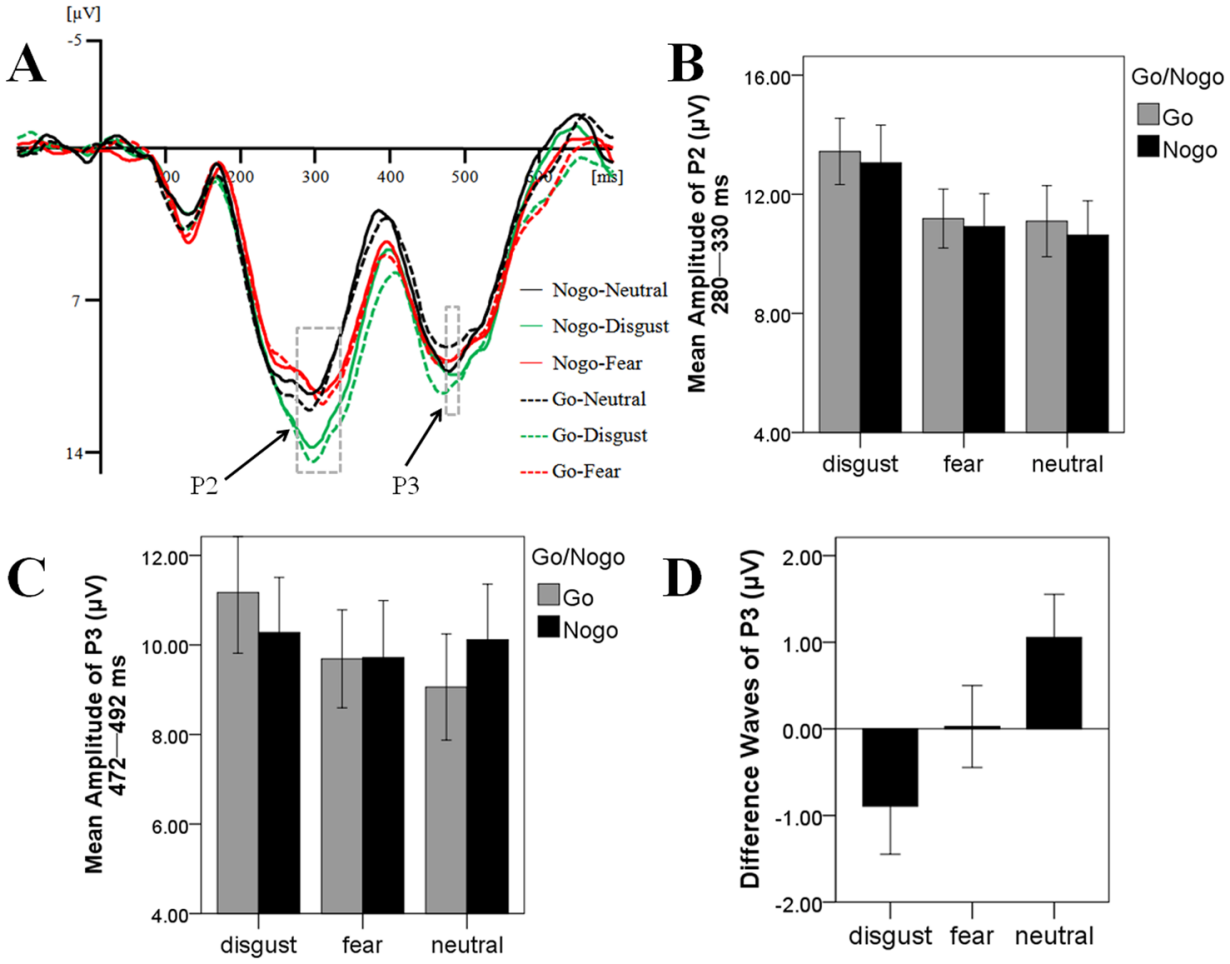
The ERP Results for Weakly Masked Condition. (A) The averaged ERP under different emotional contexts; (B) P2 amplitude under different emotional contexts; (C) P3 amplitude under different emotional contexts; (D) The difference waves (no-go condition minus go condition) of P3 amplitude under different emotional contexts.

To increase the signal-to-noise ratio, we created a region of interest (ROI) for the P2 and P3 components consisting of several centro-parietal electrodes (CPz, CP1, CP2, Pz, P1, and P2). This ROI was selected on the basis of previous studies [21,23,31]. In line with previous research, mean amplitudes of specific ERP deflections were measured for different time intervals. The time windows of the P2 and P3 components of obtained average waveforms were established based on the grand averaged potentials of each task condition. Consequently, for the strongly masked condition, the interval of the P2 component was 280–330 ms, and the interval of the P3 was 430–565 ms; for the weakly masked condition, the interval of the P2 was 280–330 ms, and the interval of the P3 was 472–492 ms (Figs 2A and 3A).

Finally, for the strongly masked condition, separate repeated-measures within-subjects ANOVAs on P2 and P3 amplitudes were firstly carried out with the factors of emotion (fear, disgust, neutral) × prime type (strongly masked go trial vs. strongly masked no-go trial). Then, the difference waves of P3 (no-go condition minus go condition) under fearful, disgusting, and neutral emotional contexts were analysed using a one-way ANOVA. For the weakly masked condition, the same analyses were carried out (When examining the ERP figures, there appears to be latency shifts in the P2 in Fig 2A, and in the P3 in Fig 3A. The latency shifts appear to occur between the fear and disgust conditions. Also, there appear to be large differences in amplitude and latency of the negative peaks (N2) that separate the two positive components. Therefore, the emotional effects on P2 latency, N2 latency and P3 latency were analysed. Besides, the correlations of P2, N2, P3 amplitude and latency under different emotional contexts were computed separately. These results were presented and reported in the S1 Appendix).

## Results

### Behavioural data

#### Probing the effectiveness of the masking procedure

For the awareness test, the accuracy was at chance level, *M* = 51.56%, *SD* = 0.09, and *d’* was not significantly different from zero, *d’* = 0.18, *SD* = 0.83, *t* (17) = 0.92, *p* = 0.37. Therefore, the prime could not be perceived in the strongly masked condition, confirming the effectiveness of the masking procedure.

#### The effect of emotional context on unconscious inhibitory control

For the strongly masked condition, the mean response accuracy was above 99%, and neither the main effects of prime type and emotion nor the interaction between prime type and emotion were significant, *p*s > 0.05.

For the RT, the results only revealed a main effect of prime type, *F* (1, 17) = 16.31, *p* = 0.01, *η*_*p*_^*2*^ = 0.49, with longer RTs for the strongly masked no-go trials (*M* = 475.33 ms, *SD* = 11.88) than for strongly masked go trials (*M* = 466.42 ms, *SD* = 11.75); therefore, it appears that unconscious inhibitory control existed in this study (Table 1). No other significant difference was observed. For the RT slowing, the one-way ANOVA revealed no significant difference, *F* (2, 53) = 0.48, *p* = 0.62.

#### The effect of emotional context on conscious inhibitory control

For the weakly masked condition, the ANOVA on response accuracy results only revealed a main effect of prime type, *F* (1, 17) = 20.01, *p* < 0.01, *η*_*p*_^*2*^ = 0.54, with higher accuracy for the weakly masked go trials (*M* = 0.99, *SD* < 0.01) than for weakly masked no-go trials (*M* = 0.93, *SD* = 0.02). No other significant difference was observed (Table 1). Moreover, the one-way ANOVA on RTs for weakly masked go trials revealed no significant difference, *F* (2, 53) = 0.30, *p* = 0.74.

### ERP data

#### The effect of emotional context on unconscious inhibitory control

The results of the ANOVA on P2 only showed a significant main effect of emotion, *F* (2, 16) = 13.30, *p <* 0.01, *η*_*p*_^*2*^ = 0.62 (Fig 2B). Post-hoc contrasts suggested that disgusting images evoked a larger P2 (*M* = 13.68 μV, *SD* = 1.28) than did fearful images (*M* = 11.21 μV, *SD* = 1.15), *t* (17) = 5.31, *p* < 0.01, and neutral images (*M* = 11.48 μV, *SD* = 0.91), *t* (17) = 3.30, *p* < 0.01. No differences were found between fearful (*M* = 11.21 μV, *SD* = 1.05) and neutral images (*M* = 11.48 μV, *SD* = 0.86), *t* (17) = -0.55, *p* = 0.59. These results are partly consistent with our hypothesis and suggest that disgusting stimuli attract and consume more attentional resources than do fearful and neutral stimuli.

The ANOVA on P3 revealed a marginally significant main effect of prime type, *F* (1, 17) = 3.28, *p* = 0.08, *η*_*p*_^*2*^ = 0.16, with a greater P3 amplitude for the strongly masked no-go trials (*M* = 7.94 μV, *SD* = 0.91) than that for the strongly masked go trials (*M* = 7.56 μV, *SD* = 0.88). This suggests the presence of unconscious inhibitory control in this study (Fig 2C).

Importantly, we found a significant interaction between emotion and prime type, *F* (2, 16) = 4.31, *p* = 0.03, *η*_*p*_^*2*^ = 0.34. Further analyses showed that, under the fearful emotional contexts, strongly masked no-go trials evoked a marginally larger P3 amplitude than did strongly masked go trials (*M* = 8.21 μV, *SD* = 3.82 vs. *M* = 7.52 μV, *SD* = 3.97), *F* (1, 17) = 4.16, *p* = 0.06, *η*_*p*_^*2*^ = 0.20. For neutral contexts, this difference was also significant (*M* = 7.68 μV, *SD* = 3.71 vs. *M* = 7.00 μV, *SD* = 3.73), *F* (1, 17) = 4.50, *p* = 0.05, *η*_*p*_^*2*^ = 0.21. However, there was no significant difference between strongly masked go and no-go trials under the disgusting contexts (*M* = 8.16 μV, *SD* = 4.02 vs. *M* = 7.91 μV, *SD* = 4.20), *F* (1, 17) = 0.74, *p* = 0.40, *η*_*p*_^*2*^ = 0.04 (Fig 2C), suggesting that disgusting stimuli may weaken unconscious inhibitory control.

The one-way ANOVA on P3 difference waves was marginally significant, *F* (2, 53) = 2.90, *p* = 0.06. Post-hoc contrasts suggested that the difference under disgusting contexts (*M* = -0.26 μV, *SD* = 0.30) was smaller than that under fearful (*M* = 0.69 μV, *SD* = 0.34), *t* (17) = -2.15, *p* = 0.05, and neutral contexts (*M* = 0.68 μV, *SD* = 0.32), *t* (17) = -2.65, *p* = 0.02. However, no difference was found between fearful (*M* = 0.69 μV, *SD* = 0.34) and neutral contexts (*M* = 0.68 μV, *SD* = 0.32), *t* (17) = 0.03, *p* = 0.97 (Fig 2D). These results are consistent with our hypothesis and demonstrate that disgusting stimuli, compared to fearful and neutral ones, impaired unconscious inhibitory control.

Finally, the correlation analysis between the P2 and the behavioral indices of unconscious inhibitory control (RT slowing and the accuracy of no-go trials) was also made to test whether unconscious inhibitory control was related directly to the extent of attentional resources available for processing. Results showed that, for the RT slowing, no significant results were observed (*ps* > 0.10). For the accuracy of no-go trials, the P2 amplitude under disgusting contexts was negatively correlated with the no-go accuracy (*r* = -0.53, *p* = 0.02). These results supported our hypothesis that disgusting distracters would consume more attentional resources and impair unconscious inhibitory control.

#### The effect of emotional context on conscious inhibitory control

The results of the ANOVA on P2 only showed a significant main effect of emotion, *F* (2, 16) = 29.81, *p <* 0.01, *η*_*p*_^*2*^ = 0.79 (Fig 3B). Post-hoc contrasts suggested that disgusting images evoked a larger P2 (*M* = 13.25 μV, *SD* = 1.14) than did fearful (*M* = 11.05 μV, *SD* = 1.04), *t* (17) = 7.42, *p* < 0.01, and neutral images (*M* = 10.86 μV, *SD* = 1.16), *t* (17) = 5.69, *p* < 0.01. No differences were found between fearful (*M* = 11.05 μV, *SD* = 1.04) and neutral images (*M* = 10.86 μV, *SD* = 1.16), *t* (17) = 0.46, *p* = 0.65. These results are partly consistent with our hypothesis and suggest that disgusting stimuli attract and consume more attentional resources than do fearful and neutral stimuli.

The ANOVA on P3 revealed a significant main effect of emotion, *F* (2, 16) = 5.19, *p* = 0.02, *η*_*p*_^*2*^ = 0.39 (Fig 3C). Post-hoc contrasts showed that the P3 under disgusting context (*M* = 10.72 μV, *SD* = 1.26) was larger than that under fearful contexts (*M* = 9.70 μV, *SD* = 1.16), *t* (17) = 3.25, *p* < 0.01, and neutral contexts (*M* = 9.59 μV, *SD* = 1.19), *t* (17) = 2.55, *p* = 0.02. However, no difference was found between fearful (*M* = 9.70 μV, *SD* = 1.16) and neutral contexts (*M* = 9.59 μV, *SD* = 1.19), *t* (17) = 0.33, *p* = 0.75.

Importantly, we found a significant interaction between emotion and prime type, *F* (2, 16) = 10.03, *p* < 0.01, *η*_*p*_^*2*^ = 0.56. Further analyses showed that, under the neutral emotional contexts, strongly masked no-go trials evoked a larger P3 amplitude than did strongly masked go trials (*M* = 10.11 μV, *SD* = 5.27 vs. *M* = 9.06 μV, *SD* = 5.03), *F* (1, 17) = 4.48, *p* = 0.05, *η*_*p*_^*2*^ = 0.21. However, there was no significant difference between strongly masked go and no-go trials under the disgusting (*M* = 11.17 μV, *SD* = 5.75 vs. *M* = 10.28 μV, *SD* = 5.21), *F* (1, 17) = 2.58, *p* = 0.13, *η*_*p*_^*2*^ = 0.13, and fearful contexts (*M* = 9.69 μV, *SD* = 4.64 vs. *M* = 9.72 μV, *SD* = 5.41), *F* (1, 17) *<* 0.01, *p* = 0.96, *η*_*p*_^*2*^ < 0.01 (Fig 3C). These results suggest that both disgusting and fearful distracters may impair conscious inhibitory control.

The one-way ANOVA on P3 difference waves was significant, *F* (2, 53) = 3.65, *p* = 0.03. Post-hoc contrasts suggested that the difference under disgusting contexts (*M* = -0.89 μV, *SD* = 0.56) was marginally smaller than that under fearful (*M* = 0.03 μV, *SD* = 0.47), *t* (17) = -1.90, *p* = 0.07, and neutral contexts (*M* = 1.06 μV, *SD* = 0.50), *t* (17) = -4.38, *p* < 0.01. Moreover, the difference under the fearful context (*M* = 0.03 μV, *SD* = 0.47) was smaller than that under the neutral context (*M* = 1.06 μV, *SD* = 0.50), *t* (17) = -2.74, *p* = 0.01 (Fig 3D). These results are consistent with our hypothesis and demonstrate that disgusting stimuli impaired conscious inhibitory control to a greater extent than did fearful stimuli.

Finally, the correlation analysis between the P2 and the behavioral indices of conscious inhibitory control (the accuracy of no-go trials) revealed no significant results (*ps* > 0.10).

## Discussion

By combining different emotional contexts with a masked Go/No-Go task, the present study examined the electrophysiological correlates of the distinct effects of fearful and disgusting distracters on inhibitory control. Consistent with our hypothesis, results showed that disgusting stimuli elicited a larger P2, and the difference waves of P3 amplitude under disgusting contexts were smaller than were those under fearful contexts for both conscious and unconscious inhibitory control. These results suggest that disgusting distracters might consume more attentional resources and therefore hinder conscious and unconscious inhibiting ability to a greater extent than do fearful distracters.

At the behavioural level, we found no significant main effect of emotion or an interaction between emotion and prime type. Nevertheless, by measuring ERPs we revealed the significant and distinct influences of fearful and disgusting distracters on both conscious and unconscious inhibitory control. To that extent, behavioural methods do not precisely reflect the differences between emotional contexts in their modulation of inhibitory control [20]. This, indeed, is one of the advantages of the ERP method-it can provide a continuous script of neural activity that leads to the response to emotional stimuli, thereby allowing for greater insight on the effects of emotional distracters on inhibitory control. Furthermore, the present results suggest that some of the behavioural strategies that are used to assess cognitive control might not be sensitive to the emotional effects on inhibitory control. It may be important for future research to utilize various assessments that include direct neurophysiological measures such as ERP or fMRI.

At the neural level, we found that disgusting and fearful distracters had different effects on attention. Specifically, we found a larger P2 for disgusting stimuli than for fearful stimuli, which suggests that disgusting stimuli attract and consume more attentional resources than fearful stimuli do [32], in line with the findings of previous studies [9,10]. The differences between fearful and disgusting stimuli could be explained by the cost-benefit principle proposed by Carretié et al. [9], who indicated that no biological costs were associated with the exploration of disgusting stimuli, even though they could signal possible danger. Instead, disgusting stimuli may turn out to be beneficial after exploration (e.g. bitter-tasting pills may have healthful effects). Therefore, when encountering disgusting stimuli, the most adaptive response is to explore them in order to clarify whether they are dangerous or beneficial. However, for fearful stimuli (e.g. predators) there are more costs than benefits involved in their exploration. As a result, more attentional resources are diverted to aid in the processing of disgusting stimuli, and a larger P2 is evoked.

More importantly, we found that disgusting distracters impaired inhibiting ability to a greater extent than fearful distracters did, as evidenced by smaller difference waves of P3 under disgusting contexts. This effect could be interpreted through the cost-benefit principle and the limitation of attentional resources [9,25]. On the one hand, as mentioned above, disgusting stimuli attract and consume more attentional resources due to their potential benefits (evidenced by the increased P2 amplitude); on the other hand, cognitive resources are limited [25]. Therefore, valuable cognitive resources are diverted to explore the disgusting stimuli, which impair subsequent inhibitory control, a process that demands resources [26,33]. As the difference between no-go and go trials is an index of inhibiting ability [12,16], it is not surprising that, in our study, the P3 difference waves under disgusting contexts were smaller than were those under fearful contexts.

In addition, we found that the disgusting distracters hinder both conscious and unconscious inhibitory control, while the fearful distracters just impede conscious inhibitory control (Figs 2D and 3D). We thought these results may relate to the differences between conscious and unconscious inhibition, as well as the differences between fear and disgust. For one thing, although both conscious and unconscious inhibition would activate prefrontal control networks [12], conscious inhibitory control is stronger in the strength, duration, and scope of neural activity [15]. The stronger neural activation may indicate that conscious inhibition needs more cognitive resources and hence might be more susceptible to the influences of resources availability. Therefore, it may be much easier to observe the impairment effects of emotional distracters on conscious inhibition since they consume limited attentional resources. For another, some researchers have indicated that the interferences of fearful distracters are smaller than disgusting distracters [9], which might be caused by the fact that compared to disgust, fear “is very difficult to generate ‘artificially’ because of its high importance in real, dangerous situations” [34]. Consequently, in the current study, the smaller interference effect of fearful distracters was only observed for conscious inhibition.

To the best of our knowledge, the current study was the first to investigate the electrophysiological correlates of the distinct effects of fearful and disgusting distracters on inhibitory control (both conscious and unconscious). The fact that fear and disgust exerted different effects on inhibitory control is important both from a methodological and a theoretical perspective. At the methodological level, it suggests that the previous exploration of negative emotional stimuli (distinguished from positive stimuli in terms of valence and arousal) in the inhibitory control literature is flawed because it inappropriately treats fear and disgust as a single category. Future research should recognize that ‘negative stimuli’ which evoke both fear and disgust might elicit mixed cognitive processes. At the theoretical level, our results suggest that in affective science, the discrete approach, which emphasizes the importance of each basic emotion [35], and the dimensional approach, which posits that emotions are a product of combinations of different valences and arousal levels [36], are both necessary and complementary for identifying the effects of emotion on executive control.

While the current study has numerous strengths, it also has some limitations that should be noted. The predictions of the current study were based on the logic that disgusting stimuli may enable greater exploration and attention toward these stimuli in contrast to fear-related stimuli. Fortunately, our results supported this hypothesis. However, as we have mentioned in the introduction, some researches have also demonstrated the suppressed sensory perceptual and attentional processing of disgust information [7,8]. As for the differences between the current study and previous study, we thought it might be related to the following reasons:

First, it might be related to the emotional images. In Krusemark and Li [8], the images used to convey fear were pictures such as spiders. However, some researchers have pointed out that the spider pictures might generate feelings of both fear and disgust and should be discarded in order to generate “pure” feeling of fear (van Hooff et al. [37]). Besides, in their study, the arousal of fearful images was higher than of disgust images. While in our current study, we excluded the pictures that might generate feelings of both fear and disgust (e.g. spiders). We also chose fearful pictures that represented more fearfulness than did disgusting and neutral pictures and disgusting pictures that represented more disgustingness than did fearful and neutral pictures, while keeping their valence and arousal ratings constant. Taken together, we thought the first potential reason for the differences between our study and Krusemark and Li [8] might be related to the emotional images. That is, the fearful images used in Krusemark and Li [8] might generate both strong feeling of fear and disgust, and the fearful images had higher level of arousal than of disgust images.

Second, it might be related to the anxiety levels of participants. The participants in Krusemark and Li [8] were selected from 563 college students based on their scores on the Behavioral Inhibition Scale (BIS, a measure for trait anxiety). Their sample consisted of 22 students with the highest scores and 21 students with the lowest scores. It should be noted that fear-related attention biases have been demonstrated most consistently in high-anxious participants. Both the behavior and ERP results of Krusemark and Li [8] also showed the same trend. Specifically, the anxious (vs. non-anxious) individuals exhibited greater search accuracy in the fear than the disgust condition (*r* = 0.36, *p* < 0.05). Also, anxiety composite scores positively correlated with differential P1 amplitude between fear and disgust conditions (*r* = 0.36, *p* = 0.02). Therefore, it could be the anxiety that contributed to the attentional bias to fearful stimuli. While in our current study, our participants might be non-anxious (unfortunately, we failed to assess the anxiety level of participants). Taken together, we thought the second potential reason for the differences between our study and Krusemark and Li [8] might be related to the anxiety levels of participants.

Third, as for the difference between our study and Susskind et al. [7], we thought it might be related to the task setting. In Susskind et al. [7], they investigated fear and disgust by the statistical model of expression appearance. But in our current study, the emotional images were tasked irrelevant and participants were informed to respond to the go/no-go signal superimposed on these images. Susskind et al. [7] showed that fear might work to enhance perception, whereas disgust dampen it, but Carretié et al. [9] proposed the cost-benefit principle, which indicated that disgusting stimuli might turn out to be beneficial after exploration. We thought these discrepancies could be reconciled. Specifically, when participants confronted with disgust stimuli, the instant response might be avoidance. But if they had to focus on these stimuli because of some reasons (e.g. task setting), the most adaptive response they might take was to explore these stimuli in order to clarify whether they are dangerous or beneficial.

Consequently, we thought the differences between our current study and previous studies might be related to the differences on emotional images, anxiety level of participant, and the task setting. Future studies should take these factors into consideration.

Some other limitations should also be noted. First, we only recruited female participants in order to exclude gender as a confounding variable [27]. Therefore, our conclusions cannot be generalized to males. Future studies comparing both genders are needed to explore whether this effect similarly exists in males. Second, previous research emphasized the close relation between fear and disgust [4]; although we tried to decompose them in our selection of stimuli, we cannot be certain that the two emotions were decomposed completely. Future research is therefore needed to explore new methods of distinguishing fear from disgust. Third, the dissociation between arousal and valence is an important issue, Verbruggen and De Houwer [2] have indicated that conscious inhibition is mainly affected by arousal and not by valence. A reasonable question, then, is whether such a conclusion also holds for unconscious inhibition. However, because the current study mainly focused on the distinct effects of fear and disgust on inhibitory control, we did not take this question into consideration. Therefore, more rigorous studies should be conducted to explore this question. Forth, since the ERP are time-locked to the onset of the go/no-go phase. Thus, the P2 will peak during different phases of the task for the different trial types. That is, it will be during the 233 ms go/no-go phase in the weakly masked condition, and during the 233 ms mask phase in the strongly masked condition. This difference seems to take away from the “conscious versus unconscious” inhibitory control argument. Consequently, more ingenious experimental designs were needed to explore this question.

## Conclusion

The present study demonstrated that fear and disgust exert different influences on inhibitory control (both conscious and unconscious). Specifically, disgusting distracters consumed more attentional resources and therefore impaired subsequent inhibitory control to a greater extent than fearful distracters did. These results expand the understanding of the relationship between emotions and inhibitory control and emphasize the importance of investigating specific categories of emotion rather than negative and positive emotion more generally.

## Supporting Information

S1 Appendix Complementary analysis on P2/N2/P3 latency and the correlations of amplitude and latency. (DOC)

S1 Table Data of the awareness test. (DOC)

S2 Table Behaviour data for the unconscious condition. (DOC)

S3 Table Behaviour data for the conscious condition. (DOC)

S4 Table P2 response for each condition. (DOC)

S5 Table P3 response for each condition. (DOC)

S6 Table Difference waves of P3 for each condition. (DOC)
