# Supplementary material for: The Divergent Effects of Fear and Disgust on Inhibitory Control: An ERP Study
Source: PLoS One. 2015 Jun 1;10(6):e0128932. doi: 10.1371/journal.pone.0128932 (PMC4452620; doi:10.1371/journal.pone.0128932)
Supplement: S1 Appendix — (DOC) [file pone.0128932.s001.doc]

**Complementary analysis:**

When examining the ERP figures, there appears to be latency shifts in the P2 in Fig. 2, and in the P3 in Fig. 3. The latency shifts appear to occur between the fear and disgust conditions (at least). Also, there appear to be large differences in amplitude and latency of the negative peaks (N2) that separate the two positive components. Therefore, the emotional effects on P2 latency, N2 latency and P3 latency were analyzed. Besides, the correlations of P2, N2, P3 amplitude and latency under different emotional contexts were computed separately (Table 1).

**ERP analysis**

The data were referenced to the average of the left and right mastoids (average mastoid reference), and a bandpass filter of 0.3–40 Hz was applied. Eye movement artefacts (such as eye movements and blinking) were excluded offline. Trials contaminated with artefacts due to amplifier clipping and peak-to-peak deflection exceeding ±70 μV were excluded from the average. Only trials with correct responses were analyzed. The continuous recording was divided into 800-ms epochs for each trial, beginning 100 ms before the go/no-go signal onset.

To increase the signal-to-noise ratio, we created a region of interest (ROI) for the P2, N2 and P3 components consisting of several centro-parietal electrodes (CPz, CP1, CP2, Pz, P1, and P2). This ROI was selected on the basis of previous studies . In addition, a computerized peak-picking program was used to measure P2, N2, and P3 peak amplitude and latency at the ROI (i.e., the time point, from 280-330 ms for P2, 360-420 ms for N2, and 430-565 ms for P3, when the voltage at ROI was maximally positive or negative).

Finally, for the strongly masked condition, the P2 latency under disgusting, fearful and neutral contexts were firstly analyzed, then the correlation of P2 amplitude and latency was analyzed. The N2 latency, N2 amplitude, the correlation of N2 amplitude and latency were analyzed using the same method. And the P3 latency, the correlation of P3 amplitude and latency were also analyzed. For the weakly masked condition, the same analyses were carried out.

**Result**

**The effect of emotional context on unconscious inhibitory control**

The results of ANOVA on P2 latency showed a significant main effect of emotion, *F* (2, 16) *=* 9.34, *p* *<* 0.01, *η_p_^2^* = 0.54. Post-hoc contrasts suggested that neutral images (*M* = 295.50 ms, *SD* = 5.55) had a shorter latency than disgusting images (*M* = 304.17 ms, *SD* = 11.97), *t* (17) *=* 3.93, *p* < 0.01, and fearful images (*M* = 305.11 ms, *SD* = 13.28), *t* (17) *=* 3.81, *p* < 0.01. No differences were found between disgusting (*M* = 304.17 ms, *SD* = 11.97) and fearful images (*M* = 305.11 ms, *SD* = 13.28), *t* (17) = -0.40, *p* = 0.70. Besides, the analysis on the correlations of P2 amplitude and latency failed to observe any significant results (see Table 1).

The results of ANOVA on N2 amplitude showed a significant main effect of emotion, *F* (2, 16) *=* 3.89, *p* *=* 0.04, *η_p_^2^* = 0.33. Post-hoc contrasts suggested that the N2 amplitude under neutral context (*M* = 3.71 μV, *SD* = 3.82) was more negative than that under disgusting context (*M* = 5.52 μV, *SD* = 4.92), *t* (17) *=* 2.65, *p* = 0.02, and fearful context (*M* = 5.24 μV, *SD* = 4.56), *t* (17) *=* 2.86, *p* = 0.01. No differences were found between disgusting (*M* = 5.52 μV, *SD* = 4.92) and fearful contexts (*M* = 5.24 μV, *SD* = 4.56), *t* (17) = 0.84, *p* = 0.41.

The results of ANOVA on N2 latency showed a significant main effect of emotion, *F* (2, 16) *=* 7.61, *p* *<* 0.01, *η_p_^2^* = 0.49. Post-hoc contrasts suggested that the N2 latency under neutral context (*M* = 388.67 ms, *SD* = 17.91) was shorter than that under disgusting context (*M* = 404.83 ms, *SD* = 18.09), *t* (17) *=* 3.78, *p* < 0.01, and fearful context (*M* = 402.94 ms, *SD* = 16.91), *t* (17) *=* 3.52, *p* < 0.01. No differences were found between disgusting (*M* = 404.83 ms, *SD* = 18.09) and fearful contexts (*M* = 402.94 ms, *SD* = 16.91), *t* (17) = 0.55, *p* = 0.59. Furthermore, the analysis on the correlations of N2 amplitude and latency failed to observe any significant results (see table 1).

The results of ANOVA on P3 latency showed no significant results, *F* (2, 16) *=* 1.00, *p* *=* 0.39, *η_p_^2^* = 0.11. Neither the the analysis on the correlations of P3 amplitude and latency reach significant (see table 1).

**The effect of emotional context on conscious inhibitory control**

The results of ANOVA on P2 latency showed a significant main effect of emotion, *F* (2, 16) *=* 4.38, *p* *=* 0.03, *η_p_^2^* = 0.35. Post-hoc contrasts suggested that fearful images (*M* = 304.72 ms, *SD* = 9.72) had a longer latency than neutral images (*M* = 295.17 ms, *SD* = 10.70), *t* (17) *=* 3.02, *p* < 0.01, and disgusting images (*M* = 297.44 ms, *SD* = 11.04), *t* (17) *=* 2.32, *p* = 0.03. No differences were found between disgusting (*M* = 297.44 ms, *SD* = 11.04) and neutral images (*M* = 295.17 ms, *SD* = 10.70), *t* (17) = 0.87, *p* = 0.40. Besides, the analysis on the correlations of P2 amplitude and latency failed to observe any significant results (see table 1).

The results of ANOVA on N2 amplitude showed a significant main effect of emotion, *F* (2, 16) *=* 8.22, *p* *<* 0.01, *η_p_^2^* = 0.51. Post-hoc contrasts suggested that the N2 amplitude under neutral context (*M* = 2.28 μV, *SD* = 5.15) was more negative than that under disgusting context (*M* = 4.75 μV, *SD* = 5.11), *t* (17) *=* 4.12, *p* < 0.01, and fearful context (*M* = 4.13 μV, *SD* = 4.53), *t* (17) *=* 3.37, *p* < 0.01. And the the N2 amplitude under fearful context (*M* = 4.13 μV, *SD* = 4.53) was smaller than that under disgusting context (*M* = 4.75 μV, *SD* = 5.11), *t* (17) *=* 2.28, *p* = 0.04.

The results of ANOVA on N2 latency showed a significant main effect of emotion, *F* (2, 16) *=* 8.27, *p* *<* 0.01, *η_p_^2^* = 0.51. Post-hoc contrasts suggested that the N2 latency under disgusting context (*M* = 403.33 ms, *SD* = 13.34) was longer than that under neutral context (*M* = 391.11 ms, *SD* = 16.79), *t* (17) *=* 3.11, *p* < 0.01, and fearful context (*M* = 395.39 ms, *SD* = 11.76), *t* (17) *=* 3.98, *p* < 0.01. No differences were found between fearful (*M* = 395.39 ms, *SD* = 11.76) and neutral context (*M* = 391.11 ms, *SD* = 16.79), *t* (17) *=* 1.25, *p* = 0.23 (see table 1). Furthermore, the analysis on the correlations of N2 amplitude and latency failed to reach significant (see table 1).

The results of ANOVA on P3 latency showed no significant results, *F* (2, 16) *=* 0.03, *p* *=* 0.97, *η_p_^2^* < 0.01. Neither the analysis on the correlations of P3 amplitude and latency reach significant (except the correlation under disgusting context: *r* = -0.51, *p* = 0.03).

**Discussion**

Taken together, the results demonstrated the impairment effect of negative stimuli on conflict monitoring. Specifically, we found that the P2 latency under emotional contexts were longer than that under neutral contexts. Moreover, the amplitude of N2 under emotional contexts was smaller than that under neutral contexts. Besides, the latency of N2 under emotional contexts was longer than that under neutral contexts. These results were consistent with previous studies (Huang & Luo, 2006; Xin et al., 2010; Yu et al., 2009; Yang et al., 2014). On the one hand, the slower P2 under emotional contexts might indicate that people would prefer to avoid the threat stimuli and therefore resulted in the less efficient processing of visual information at a relatively early stage, it might also indicated that the emotional stimuli requie longer attentional engagement due to a greater informational burden than neitral stimuli (Huang & Luo, 2006). On the other hand, the smaller and slower N2 under emotional contexts might indicate that the negative stimuli would consume more attentional resource and therefore fewer resources were left for the conflict monitoring. Future studies were needed to certify this hypothesis and replicate these results.

Huang, Y. X., & Luo, Y. J. (2006). Temporal course of emotional negativity bias: an ERP study. *Neuroscience letters*, *398*(1), 91-96.

Xin Y, Li H, Yuan J-J. (2010). Negative Emotion Interferes with Behavioral Inhibitory Control: An ERP Study. Acta Psychologica Sinica 42: 334-341.

Yu, F., Yuan, J., & Luo, Y. J. (2009). Auditory-induced emotion modulates processes of response inhibition: an event-related potential study.NeuroReport, 20(1), 25-30.

Yang, J., Qi, M., & Guan, L. (2014). Self-esteem modulates the latency of P2 component in implicit self-relevant processing. *Biological psychology*, *97*, 22-26.

Table 1. Amplitude, latency, and correlation of amplitude and latency of P2, N2, and P3 for each condition.

| P2 | | | |
| --- | --- | --- | --- |
|  | Disgust | Fear | Neutral |
| Strongly masked condition |  |  |  |
| Amplitude | 14.71 (5.40) | 12.16 (5.25) | 12.94 (4.32) |
| Latency | 304.17 (11.97) | 305.11 (13.28) | 295.50 (5.55) |
| Correlation of amplitude and latency | 0.26 | 0.32 | 0.12 |
|  |  |  |  |
| Weakly masked condition |  |  |  |
| Amplitude | 14.53 (5.51) | 11.93 (4.63) | 12.30 (4.98) |
| Latency | 297.44 (11.04) | 304.72 (9.72) | 295.17 (10.70) |
| Correlation of amplitude and latency | 0.04 | 0.16 | 0.18 |
|  |  |  |  |
| N2 | | | |
| Strongly masked condition |  |  |  |
| Amplitude | 5.52 (4.92) | 5.24 (4.56) | 3.71 (3.82) |
| Latency | 404.83 (18.09) | 402.94 (16.91) | 388.67 (17.91) |
| Correlation of amplitude and latency | -0.14 | -0.13 | 0.41 |
|  |  |  |  |
| Weakly masked condition |  |  |  |
| Amplitude | 4.75 (5.11) | 4.13 (4.53) | 2.28 (5.15) |
| Latency | 403.33 (13.34) | 395.39 (11.76) | 391.11 (16.79) |
| Correlation of amplitude and latency | -0.18 | -0.34 | -0.14 |
|  |  |  |  |
| P3 | | | |
| Strongly masked condition |  |  |  |
| Amplitude | 10.21 (4.97) | 9.85 (4.82) | 9.29 (4.13) |
| Latency | 477.11 (22.98) | 482.67 (26.32) | 482.56 (21.93) |
| Correlation of amplitude and latency | -0.51* | -0.33 | -0.24 |
|  |  |  |  |
| Weakly masked condition |  |  |  |
| Amplitude | 11.97 (5.51) | 10.93 (5.15) | 10.46 (5.19) |
| Latency | 484.61 (23.06) | 484.17 (23.58) | 483.72 (20.88) |
| Correlation of amplitude and latency | -0.42 | -0.38 | -0.32 |
