# Supplementary material for: The Divergent Effects of Fear and Disgust on Inhibitory Control: An ERP Study
Source: PLoS One. 2015 Jun 1;10(6):e0128932. doi: 10.1371/journal.pone.0128932 (PMC4452620; doi:10.1371/journal.pone.0128932)
Supplement: S1 Table — (DOC) [file pone.0128932.s002.doc]

| ID | Correct percentage | Hit rate | Z of hit rate | False rate | Z of false rate | d^，^ score |
| --- | --- | --- | --- | --- | --- | --- |
| 1 | 0.45 | 0.43 | -0.18 | 0.53 | 0.08 | -0.25 |
| 2 | 0.42 | 0.30 | -0.52 | 0.47 | -0.08 | -0.45 |
| 3 | 0.55 | 0.50 | 0.00 | 0.40 | -0.25 | 0.25 |
| 4 | 0.79 | 0.57 | 0.18 | 0.00 | -3.09 | 3.27 |
| 5 | 0.60 | 0.53 | 0.08 | 0.33 | -0.44 | 0.52 |
| 6 | 0.54 | 0.47 | -0.08 | 0.40 | -0.25 | 0.18 |
| 7 | 0.50 | 0.43 | -0.18 | 0.43 | -0.18 | 0.00 |
| 8 | 0.47 | 0.50 | 0.00 | 0.57 | 0.18 | -0.18 |
| 9 | 0.60 | 0.67 | 0.44 | 0.47 | -0.08 | 0.52 |
| 10 | 0.42 | 0.40 | -0.25 | 0.57 | 0.18 | -0.43 |
| 11 | 0.53 | 0.43 | -0.18 | 0.37 | -0.33 | 0.16 |
| 12 | 0.45 | 0.37 | -0.33 | 0.47 | -0.08 | -0.26 |
| 13 | 0.57 | 0.67 | 0.44 | 0.53 | 0.08 | 0.36 |
| 14 | 0.57 | 0.60 | 0.25 | 0.47 | -0.08 | 0.33 |
| 15 | 0.42 | 0.40 | -0.25 | 0.57 | 0.18 | -0.43 |
| 16 | 0.45 | 0.40 | -0.25 | 0.50 | 0.00 | -0.25 |
| 17 | 0.50 | 0.47 | -0.08 | 0.47 | -0.08 | 0.00 |
| 18 | 0.49 | 0.40 | -0.25 | 0.43 | -0.18 | -0.08 |

S1 Table. Data of the awareness test.

Note:

“ID” means “identification of participants”;

"Correct percentage" means "mean correct response percentage”;

"Hit rate" means "hit rate";

"Z of hit rate" means "Z score of hit rate";

"False rate" means "false rate";

"Z of false rate" means "Z score of false rate";

"d^，^ score" is equal to "Z score of hit rate minus Z score of false rate".
