# Supplementary material for: The Divergent Effects of Fear and Disgust on Inhibitory Control: An ERP Study
Source: PLoS One. 2015 Jun 1;10(6):e0128932. doi: 10.1371/journal.pone.0128932 (PMC4452620; doi:10.1371/journal.pone.0128932)
Supplement: S2 Table — (DOC) [file pone.0128932.s003.doc]

S2 Table. Behaviour data for the unconscious condition.

| ID | Mean RT (ms) | | | | | | RT slowing | | | ACC | | | | | |
| --- | --- | --- | --- | --- | --- | --- | --- | --- | --- | --- | --- | --- | --- | --- | --- |
|  | disgust-go | disgust-nogo | fear-go | fear-nogo | neutral-go | neutral-nogo | disgust | fear | neutral | disgust-go | disgust-nogo | fear-go | fear-nogo | neutral-go | neutral-nogo |
| 1 | 395.54 | 399.02 | 390.50 | 402.32 | 386.24 | 392.44 | 3.48 | 11.82 | 6.20 | 1.00 | 1.00 | 1.00 | 1.00 | 1.00 | 1.00 |
| 2 | 479.06 | 487.90 | 479.48 | 490.16 | 485.04 | 488.58 | 8.84 | 10.68 | 3.54 | 1.00 | 1.00 | 1.00 | 1.00 | 1.00 | 1.00 |
| 3 | 520.24 | 495.06 | 487.58 | 482.34 | 470.21 | 477.16 | -25.18 | -5.24 | 6.95 | 1.00 | 0.98 | 1.00 | 1.00 | 0.94 | 0.98 |
| 4 | 463.44 | 467.90 | 458.68 | 457.10 | 479.62 | 480.68 | 4.46 | -1.58 | 1.06 | 1.00 | 1.00 | 1.00 | 1.00 | 1.00 | 1.00 |
| 5 | 434.54 | 462.48 | 452.88 | 486.86 | 457.08 | 472.24 | 27.94 | 33.98 | 15.16 | 1.00 | 1.00 | 1.00 | 1.00 | 1.00 | 1.00 |
| 6 | 457.70 | 494.66 | 469.02 | 487.24 | 464.58 | 478.78 | 36.96 | 18.22 | 14.20 | 1.00 | 1.00 | 1.00 | 1.00 | 1.00 | 1.00 |
| 7 | 429.24 | 432.78 | 421.16 | 436.04 | 424.60 | 428.88 | 3.54 | 14.88 | 4.28 | 1.00 | 1.00 | 1.00 | 1.00 | 1.00 | 1.00 |
| 8 | 466.60 | 452.58 | 465.60 | 465.82 | 468.06 | 470.64 | -14.02 | 0.22 | 2.58 | 1.00 | 1.00 | 1.00 | 1.00 | 1.00 | 1.00 |
| 9 | 451.96 | 442.16 | 454.74 | 453.10 | 436.49 | 474.66 | -9.80 | -1.64 | 38.17 | 1.00 | 1.00 | 1.00 | 0.98 | 1.00 | 1.00 |
| 10 | 581.65 | 588.50 | 582.44 | 613.14 | 585.74 | 580.74 | 6.85 | 30.70 | -5.00 | 1.00 | 1.00 | 1.00 | 1.00 | 1.00 | 1.00 |
| 11 | 479.90 | 492.76 | 466.96 | 504.58 | 504.00 | 496.98 | 12.86 | 37.62 | -7.02 | 1.00 | 1.00 | 1.00 | 1.00 | 1.00 | 0.98 |
| 12 | 529.43 | 526.75 | 514.27 | 542.45 | 524.48 | 530.81 | -2.68 | 28.18 | 6.33 | 1.00 | 1.00 | 1.00 | 1.00 | 1.00 | 0.94 |
| 13 | 448.40 | 455.08 | 461.34 | 461.56 | 469.08 | 462.44 | 6.68 | 0.22 | -6.64 | 1.00 | 1.00 | 1.00 | 1.00 | 1.00 | 1.00 |
| 14 | 487.54 | 476.32 | 482.98 | 488.12 | 484.32 | 499.92 | -11.22 | 5.14 | 15.60 | 1.00 | 1.00 | 1.00 | 1.00 | 1.00 | 1.00 |
| 15 | 368.82 | 382.24 | 382.12 | 368.36 | 370.24 | 408.02 | 13.42 | -13.76 | 37.78 | 1.00 | 1.00 | 1.00 | 1.00 | 1.00 | 1.00 |
| 16 | 534.46 | 543.02 | 523.54 | 556.40 | 543.58 | 537.68 | 8.56 | 32.86 | -5.90 | 1.00 | 1.00 | 1.00 | 1.00 | 1.00 | 1.00 |
| 17 | 429.44 | 484.08 | 465.80 | 479.33 | 462.39 | 470.59 | 54.64 | 13.53 | 8.20 | 1.00 | 0.98 | 1.00 | 0.98 | 0.94 | 0.98 |
| 18 | 408.90 | 413.32 | 431.56 | 429.70 | 413.46 | 415.08 | 4.42 | -1.86 | 1.62 | 1.00 | 1.00 | 1.00 | 1.00 | 1.00 | 1.00 |

Note:

“ID” means “identification of participants”;
