# Supplementary material for: The Divergent Effects of Fear and Disgust on Inhibitory Control: An ERP Study
Source: PLoS One. 2015 Jun 1;10(6):e0128932. doi: 10.1371/journal.pone.0128932 (PMC4452620; doi:10.1371/journal.pone.0128932)
Supplement: S3 Table — (DOC) [file pone.0128932.s004.doc]

| ID | Mean RT (ms) | | | ACC | | | | | |
| --- | --- | --- | --- | --- | --- | --- | --- | --- | --- |
|  | disgust | fear | neutral | disgust-go | disgust-nogo | fear-go | fear-nogo | neutral-go | neutral-nogo |
| 1 | 452.34 | 449.88 | 429.20 | 1.00 | 0.94 | 1.00 | 0.98 | 1.00 | 0.76 |
| 2 | 558.92 | 545.04 | 551.72 | 1.00 | 1.00 | 1.00 | 1.00 | 1.00 | 0.98 |
| 3 | 500.62 | 463.5 | 465.94 | 1.00 | 0.92 | 1.00 | 0.82 | 1.00 | 0.84 |
| 4 | 520.72 | 513.34 | 512.22 | 1.00 | 0.96 | 1.00 | 0.88 | 1.00 | 1.00 |
| 5 | 537.98 | 523.30 | 486.14 | 1.00 | 0.9 | 1.00 | 0.96 | 1.00 | 0.94 |
| 6 | 497.56 | 499.76 | 503.86 | 1.00 | 0.98 | 1.00 | 1.00 | 1.00 | 0.96 |
| 7 | 498.28 | 461.04 | 475.72 | 1.00 | 0.96 | 1.00 | 0.94 | 1.00 | 0.92 |
| 8 | 599.06 | 588.20 | 590.82 | 1.00 | 0.94 | 1.00 | 0.94 | 1.00 | 0.96 |
| 9 | 527.65 | 502.18 | 508.84 | 1.00 | 0.86 | 1.00 | 0.92 | 1.00 | 0.86 |
| 10 | 648.46 | 621.34 | 640.50 | 1.00 | 1.00 | 1.00 | 1.00 | 1.00 | 1.00 |
| 11 | 539.54 | 557.54 | 554.22 | 1.00 | 0.96 | 1.00 | 0.94 | 1.00 | 0.94 |
| 12 | 560.53 | 561.85 | 558.31 | 1.00 | 0.88 | 0.98 | 0.88 | 0.98 | 0.88 |
| 13 | 524.46 | 519.82 | 522.28 | 1.00 | 1.00 | 1.00 | 0.98 | 1.00 | 0.98 |
| 14 | 558.92 | 551.74 | 542.40 | 1.00 | 0.98 | 1.00 | 0.98 | 1.00 | 0.96 |
| 15 | 503.88 | 485.04 | 475.22 | 1.00 | 0.94 | 1.00 | 0.94 | 1.00 | 0.96 |
| 16 | 608.27 | 600.22 | 599.44 | 1.00 | 0.94 | 1.00 | 0.96 | 1.00 | 0.96 |
| 17 | 507.11 | 489.20 | 527.62 | 0.96 | 0.72 | 0.98 | 0.62 | 0.94 | 0.7 |
| 18 | 480.46 | 490.54 | 476.58 | 1.00 | 0.94 | 1.00 | 0.94 | 1.00 | 1.00 |

S3 Table. Behaviour data for the conscious condition.

Note:

“ID” means “identification of participants”;
