# Supplementary material for: The Divergent Effects of Fear and Disgust on Inhibitory Control: An ERP Study
Source: PLoS One. 2015 Jun 1;10(6):e0128932. doi: 10.1371/journal.pone.0128932 (PMC4452620; doi:10.1371/journal.pone.0128932)
Supplement: S4 Table — (DOC) [file pone.0128932.s005.doc]

| ID | unconscious condition (μV) | | | | | | conscious condition (μV) | | | | | |
| --- | --- | --- | --- | --- | --- | --- | --- | --- | --- | --- | --- | --- |
|  | disgust-go | disgust-nogo | fear-go | fear-nogo | neutral-go | neutral-nogo | disgust-go | disgust-nogo | fear-go | fear-  nogo | neutral-go | neutral-nogo |
| 1 | 4.58 | 3.47 | 1.21 | 0.71 | 4.16 | 5.17 | 4.93 | 4.26 | 3.57 | 2.42 | 3.46 | 2.16 |
| 2 | 16.86 | 16.82 | 14.61 | 12.9 | 14.66 | 18.60 | 16.44 | 16.06 | 14.44 | 15.07 | 17.12 | 15.88 |
| 3 | 20.69 | 19.06 | 16.34 | 14.56 | 15.77 | 14.62 | 19.97 | 17.78 | 14.68 | 14.37 | 17.14 | 13.79 |
| 4 | 11.88 | 12.03 | 8.72 | 8.17 | 8.85 | 8.10 | 11.93 | 8.96 | 8.32 | 6.40 | 8.07 | 6.20 |
| 5 | 7.82 | 8.23 | 4.35 | 7.28 | 8.32 | 7.65 | 6.97 | 4.85 | 6.02 | 4.82 | 6.20 | 4.30 |
| 6 | 15.91 | 15.42 | 11.88 | 10.23 | 12.87 | 14.37 | 15.80 | 16.18 | 12.02 | 11.21 | 11.88 | 12.71 |
| 7 | 13.20 | 15.11 | 11.43 | 12.13 | 8.14 | 12.71 | 15.54 | 12.57 | 12.65 | 11.83 | 11.22 | 11.4 |
| 8 | 12.16 | 13.61 | 14.26 | 13.16 | 12.46 | 13.09 | 12.86 | 12.69 | 12.55 | 11.17 | 13.66 | 11.96 |
| 9 | 9.71 | 11.91 | 6.97 | 7.29 | 9.64 | 10.18 | 10.05 | 10.26 | 7.90 | 8.56 | 8.83 | 8.79 |
| 10 | 26.80 | 24.38 | 18.74 | 17.68 | 17.54 | 10.51 | 13.55 | 23.40 | 14.94 | 16.07 | 17.33 | 18.59 |
| 11 | 11.19 | 9.50 | 7.24 | 8.66 | 7.19 | 7.56 | 12.23 | 11.21 | 10.75 | 8.77 | 3.76 | 5.41 |
| 12 | 9.98 | 9.58 | 6.75 | 9.57 | 9.27 | 10.45 | 8.48 | 8.69 | 6.35 | 9.62 | 7.47 | 6.59 |
| 13 | 8.91 | 10.11 | 9.72 | 10.66 | 9.49 | 8.98 | 10.77 | 10.62 | 10.47 | 9.63 | 7.46 | 7.63 |
| 14 | 14.16 | 13.59 | 11.37 | 13.94 | 11.92 | 14.55 | 16.73 | 14.49 | 13.81 | 13.22 | 13.92 | 11.83 |
| 15 | 9.67 | 8.97 | 6.07 | 7.35 | 7.83 | 8.31 | 9.87 | 10.12 | 7.07 | 7.44 | 7.26 | 8.36 |
| 16 | 17.42 | 16.66 | 15.19 | 17.83 | 14.82 | 15.43 | 18.67 | 17.18 | 15.92 | 17.14 | 16.62 | 16.40 |
| 17 | 23.99 | 22.82 | 20.14 | 21.23 | 19.92 | 20.03 | 23.66 | 23.76 | 20.27 | 21.22 | 20.00 | 18.95 |
| 18 | 13.85 | 12.54 | 14.02 | 11.21 | 10.05 | 10.20 | 13.39 | 11.97 | 9.56 | 7.48 | 8.34 | 10.39 |

S4 Table. P2 response for each condition.
