# Supplementary material for: The Divergent Effects of Fear and Disgust on Inhibitory Control: An ERP Study
Source: PLoS One. 2015 Jun 1;10(6):e0128932. doi: 10.1371/journal.pone.0128932 (PMC4452620; doi:10.1371/journal.pone.0128932)
Supplement: S5 Table — (DOC) [file pone.0128932.s006.doc]

| ID | unconscious condition (μV) | | | | | | conscious condition (μV) | | | | | |
| --- | --- | --- | --- | --- | --- | --- | --- | --- | --- | --- | --- | --- |
|  | disgust-go | disgust-nogo | fear-go | fear-  nogo | neutral-go | neutral-nogo | disgust-go | disgust-nogo | fear-go | fear-  nogo | neutral-go | neutral-nogo |
| 1 | 1.66 | 0.80 | 1.14 | 1.70 | 1.16 | 1.12 | 0.85 | 2.66 | 0.87 | 2.05 | 1.80 | 2.37 |
| 2 | 5.62 | 7.22 | 7.35 | 5.96 | 7.62 | 9.43 | 13.54 | 10.63 | 12.60 | 14.20 | 14.36 | 14.11 |
| 3 | 5.24 | 5.21 | 6.96 | 6.32 | 3.92 | 5.16 | 14.9 | 10.85 | 12.00 | 10.47 | 12.93 | 11.29 |
| 4 | 8.33 | 8.22 | 7.52 | 6.18 | 5.54 | 6.97 | 9.72 | 6.05 | 7.21 | 4.18 | 8.39 | 6.18 |
| 5 | 7.19 | 6.96 | 5.17 | 7.59 | 8.35 | 7.44 | 4.77 | 2.90 | 4.07 | 4.07 | 5.08 | 5.17 |
| 6 | 10.04 | 11.12 | 11.97 | 11.40 | 8.94 | 10.99 | 19.73 | 16.78 | 18.1 | 18.53 | 15.95 | 15.99 |
| 7 | 13.67 | 13.30 | 9.60 | 11.99 | 6.94 | 11.09 | 15.68 | 14.49 | 12.27 | 9.90 | 11.41 | 11.10 |
| 8 | 10.05 | 11.85 | 11.93 | 12.68 | 8.81 | 10.22 | 13.65 | 13.20 | 12.85 | 13.85 | 12.09 | 13.81 |
| 9 | 2.14 | 0.93 | 0.65 | 1.57 | 0.70 | 1.46 | 1.67 | 1.31 | 2.14 | 1.21 | 0.28 | 1.73 |
| 10 | 18.13 | 16.11 | 15.27 | 14.95 | 15.1 | 13.5 | 20.73 | 18.86 | 16.35 | 16.89 | 16.58 | 18.10 |
| 11 | 5.74 | 3.09 | 4.01 | 4.76 | 1.87 | 1.59 | 9.40 | 6.58 | 7.54 | 4.91 | 1.25 | 2.16 |
| 12 | 7.20 | 7.16 | 4.03 | 6.91 | 6.92 | 7.79 | 4.66 | 8.50 | 6.66 | 6.33 | 6.07 | 6.83 |
| 13 | 5.36 | 6.47 | 5.80 | 7.62 | 6.57 | 6.60 | 6.44 | 6.62 | 6.00 | 5.15 | 6.12 | 6.07 |
| 14 | 9.47 | 9.81 | 9.04 | 11.17 | 9.33 | 9.32 | 14.81 | 10.26 | 12.15 | 9.96 | 12.17 | 11.13 |
| 15 | 5.44 | 3.49 | 4.03 | 4.44 | 3.80 | 3.98 | 9.58 | 10.65 | 8.73 | 10.71 | 5.34 | 10.56 |
| 16 | 11.52 | 11.70 | 10.60 | 10.26 | 11.6 | 10.35 | 14.56 | 16.33 | 12.49 | 16.84 | 11.46 | 15.90 |
| 17 | 9.79 | 10.04 | 8.54 | 11.42 | 10.19 | 11.23 | 15.20 | 16.54 | 11.93 | 15.05 | 12.75 | 15.94 |
| 18 | 10.44 | 8.95 | 11.78 | 10.91 | 8.67 | 9.95 | 11.18 | 11.79 | 10.45 | 10.59 | 9.04 | 13.63 |

S5 Table. P3 response for each condition.
