# Supplementary material for: The Divergent Effects of Fear and Disgust on Inhibitory Control: An ERP Study
Source: PLoS One. 2015 Jun 1;10(6):e0128932. doi: 10.1371/journal.pone.0128932 (PMC4452620; doi:10.1371/journal.pone.0128932)
Supplement: S6 Table — (DOC) [file pone.0128932.s007.doc]

| ID | unconscious condition (μV) | | | conscious condition (μV) | | |
| --- | --- | --- | --- | --- | --- | --- |
|  | disgust | fear | neutral | disgust | fear | neutral |
| 1 | -0.87 | 0.55 | -0.04 | 1.80 | 1.18 | 0.57 |
| 2 | 1.60 | -1.38 | 1.81 | -2.90 | 1.60 | -0.26 |
| 3 | -0.02 | -0.64 | 1.24 | -4.06 | -1.53 | -1.64 |
| 4 | -0.11 | -1.34 | 1.43 | -3.67 | -3.03 | -2.21 |
| 5 | -0.24 | 2.42 | -0.90 | -1.87 | 0.01 | 0.09 |
| 6 | 1.09 | -0.57 | 2.05 | -2.95 | 0.43 | 0.04 |
| 7 | -0.37 | 2.39 | 4.15 | -1.19 | -2.38 | -0.31 |
| 8 | 1.80 | 0.76 | 1.41 | -0.45 | 1.00 | 1.72 |
| 9 | -1.21 | 0.92 | 0.76 | -0.36 | -0.93 | 1.45 |
| 10 | -2.01 | -0.32 | -1.60 | -1.87 | 0.54 | 1.51 |
| 11 | -2.65 | 0.75 | -0.28 | -2.81 | -2.63 | 0.91 |
| 12 | -0.04 | 2.88 | 0.87 | 3.84 | -0.33 | 0.76 |
| 13 | 1.12 | 1.82 | 0.03 | 0.17 | -0.85 | -0.05 |
| 14 | 0.34 | 2.13 | -0.01 | -4.55 | -2.19 | -1.04 |
| 15 | -1.95 | 0.42 | 0.19 | 1.07 | 1.98 | 5.22 |
| 16 | 0.18 | -0.34 | -1.25 | 1.77 | 4.34 | 4.44 |
| 17 | 0.25 | 2.88 | 1.04 | 1.34 | 3.13 | 3.20 |
| 18 | -1.49 | -0.87 | 1.28 | 0.61 | 0.14 | 4.59 |

S6 Table. Difference waves of P3 for each condition.
